# Developing a national plan for rare diseases in Germany through concerted action: the national action league for people with rare diseases

**DOI:** 10.1186/1750-1172-7-S2-A9

**Published:** 2012-11-22

**Authors:** Véronique Héon-Klin, Alexandra Halbach, Miriam Schlangen, Birgit Schnieders

**Affiliations:** 1 Federal Ministry of Health, Germany; 2 Coordination Office of national action league for people with rare diseases – Nationales Aktionsbündnis für Menschen mit seltenen Erkrankungen (NAMSE) c/o Mukoviszidose Institut gGmbH, Germany

## Introduction

To improve health and well-being of people with rare diseases and to implement the Council Recommendation of the European Union on rare diseases the German Federal Ministry of Health, in cooperation with the Federal Ministry of Education and Research and the National Alliance of Patient Groups for Rare Diseases, has initiated a national action league for people with rare diseases - Nationales Aktionsbündnis für Menschen mit Seltenen Erkrankungen (NAMSE). NAMSE brings together all key bodies and organisations of the German health care system (27 in total) to enable concerted action based on the adoption of a joint declaration. In four working groups on “Information, Diagnosis, Care/Centres/Networks and Research” of NAMSE recommendations are being developed.

## Results

**Figure 1:**
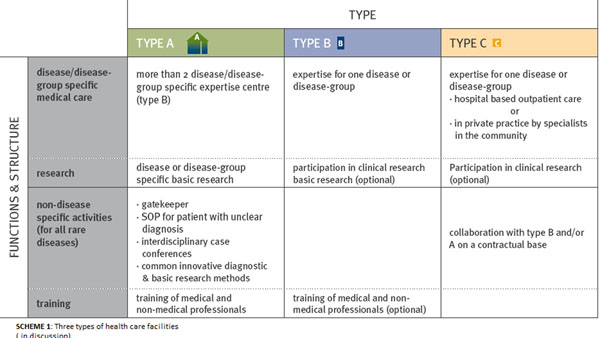


The process of identifying and later labelling **national centres** of expertise and their participation in European Reference Networks is of central importance. Three types of centres have already been defined on the basis of specific criteria (Figure 1). These can be differentiated in disease/disease-group specific medical care functions or structures and non-disease specific activities (important for all rare diseases). To guide patients and health care professionals through the health care system a common, quality assured information platform, pooling the existing information services, is discussed.

NAMSE pursues a patient-centred approach respecting the patients and their concerns. Therefore **seamless care pathways** are a recurrent theme throughout the planned measures. They are considered to accelerate the diagnosis and are of central importance for patient without diagnosis.

## Conclusions

Each working group of NAMSE has developed a set of advices. For all special indicators with targets and a timeline are being developed to evaluate their effectiveness after their implementation. Therefore an evaluation board comments all indicators proposed for the different advices. All advices have to be prioritised based on their probability of implementation by a consensus conference. The draft of German National Action Plan will be assigned to the Federal Ministry of Health in 2013.

